# L-asparaginase inhibits invasive and angiogenic activity and induces autophagy in ovarian cancer

**DOI:** 10.1111/j.1582-4934.2012.01547.x

**Published:** 2012-09-26

**Authors:** Minshu Yu, Ryan Henning, Amanda Walker, Geoffrey Kim, Alyssa Perroy, Riccardo Alessandro, Victoria Virador, Elise C Kohn

**Affiliations:** a Molecular Signaling Section Medical Oncology Branch Center for Cancer Research, National Cancer InstituteBethesda, MD, USA; b Dipartimento di Biopatologia e Biotecnologie Mediche e Forensi Sezione di Biologia e Genetica, Università di PalermoPalermo, Italy

**Keywords:** asparaginase, ovarian cancer, sialyl Lewis X, angiogenesis, autophagy

## Abstract

Recent work identified L-asparaginase (L-ASP) as a putative therapeutic target for ovarian cancer. We suggest that L-ASP, a dysregulator of glycosylation, would interrupt the local microenvironment, affecting the ovarian cancer cell—endothelial cell interaction and thus angiogenesis without cytotoxic effects. Ovarian cancer cell lines and human microvascular endothelial cells (HMVEC) were exposed to L-ASP at physiologically attainable concentrations and subjected to analyses of endothelial tube formation, invasion, adhesion and the assessment of sialylated proteins involved in matrix-associated and heterotypic cell adhesion. Marked reduction in HMVEC tube formation *in vitro*, HMVEC and ovarian cancer cell invasion, and heterotypic cell-cell and cell-matrix adhesion was observed (*P* < 0.05–0.0001). These effects were associated with reduced binding to ß1integrin, activation of FAK, and cell surface sialyl Lewis^X^ (sLe^x^) expression. No reduction in HMVEC E-selectin expression was seen consistent with the unidirectional inhibitory actions observed. L-ASP concentrations were non-toxic to either ovarian cancer or HMVEC lines in the time frame of the assays. However, early changes of autophagy were observed in both cell types with induction of ATG12, beclin-1, and cleavage of LC-3, indicating cell injury did occur. These data and the known mechanism of action of L-ASP on glycosylation of nascent proteins suggest that L-ASP reduces of ovarian cancer dissemination and progression through modification of its microenvironment. The reduction of ovarian cancer cell surface sLe^x^ inhibits interaction with HMVEC and thus HMVEC differentiation into tubes, inhibits interaction with the local matrix reducing invasive behaviour, and causes cell injury initiating autophagy in tumour and vascular cells.

## Introduction

Tumour neovascularization and invasion are early and obligate events dependent upon expression of cell surface glycoproteins and glycoconjugates [1–4]. Sialyl Lewis^X^ (sLe^x^) is a tetrasaccharide carbohydrate that is often linked to cell-surface glycoproteins. Sialic acids in general are overexpressed in some tumour cell types and play important roles in biological characteristics of cancer and other cells that are implicated in cellular invasiveness, differentiation and tumourigenecity [5–7]. L-asparaginase (L-ASP) has been reported to prevent several forms of glycosylation, including sialylation, of newly synthesized proteins [8–10].

The antimetabolic activity of L-ASP against acute lymphocytic leukaemia (ALL) has been related to degradation and loss of the essential amino acid, asparagine, in this rapidly growing and highly bioactive cancer [8, 10, 11]. The lower metabolic activity and slower proliferation rate of ovarian cancers compared with ALL argues against an essential amino acid-based anti-metabolic activity. Lorenzi *et al*. demonstrated L-ASP inhibited growth of some epithelial ovarian cancer cell lines and correlated growth inhibition with–omics findings implicating low expression of the enzyme asparagine synthetase (ASNS), that breaks down L-ASP, in a fraction of ovarian cancer patients [12, 13]. That activity was due to protection of the L-ASP. We reasoned that the altered glycosylation events caused by L-ASP would cause some of the injurious effects of L-ASP and examined effects on invasive and cell- and matrix- interactions of ovarian cancer cells.

Sialyl Le^x^ is a major E-selectin ligand, mediating both homotypic and heterotypic cell adhesion of endothelial cells [14–16]. Endothelial cell surface E-selectin was shown to be necessary for endothelial cell (EC) binding to colon cancer cells [14]. Its presence on the surface of ECs is at least partly responsible for their morphogenesis into capillary-like tubes *in vitro* [16]. Interactions between the tumour cell and the ECM play an equally important role in the initiation of angiogenesis and invasion, and to support survival [14, 17–19]. These interactions are primarily activated by integrins, a family of heavily glycosylated cell surface proteins [20, 21]. Integrin activation signals through critical survival and invasion pathways that are mediated by focal adhesion kinase (FAK) [19, 22–24]. Inhibition of integrin binding and function results in decreased angiogenic and invasive properties of cancer cells and loss of matrix-independent growth, a process called anoikis [25, 26]. Numerous studies indicate that N-linked glycosylation expression patterns of integrins are crucial to their ability to recognize extracellular matrix proteins and thus support these cellular events [27–30].

Attempts to use L-ASP in the treatment of solid malignancies were made in the 1970's without remarkable effect. Advances in solid tumour management have led to improved patient clinical status and allowed reconsideration of older drugs that may have useful mechanisms of action. Ovarian cancer has a unique vulnerability to agents that target signalling between the tumour and its local environment [31–33] and is thus an ideal model system in which to evaluate the ability of L-ASP to alter the tumour microenvironment. We suggest that by altering the expression of cell-surface glycoproteins and glycoconjugates, L-ASP will significantly alter the interactions between microvascular ECs, ovarian cancer cells, and ECM components, resulting also in ovarian cancer cell injury.

## Materials and methods

### Materials

The VEGF_165_ was from R&D (Minneapolis, MN, USA). Matrigel and Biocoat Matrigel invasion chambers were purchased from BD Biosciences (Bedford, MA, USA). XTT reagent was from Roche (Indianapolis, IN, USA). Zymogram and immunoblot gels were from Invitrogen (Minneapolis, MN, USA). L-ASP and all other materials were reagent or molecular grade. DMSO 0.1% was the vehicle control in all experiments.

### Cells and viability

Several ovarian cancer cell lines were used to evaluate the potential generalizability of the findings. Human ovarian cancer cell lines were obtained from the ATCC (Manassas, VA, USA) and HEYA8 cells were a gift of Dr. G. Mills (MD Anderson Cancer Center, Houston, TX, USA; all were validated within 6 months of use). They were maintained in RPMI-1640 medium supplemented with 5% or 10% foetal bovine serum and no more than 15% loss of viability was observed in each of the cell lines with concentrations up to 3 U/ml and continuous exposure duration up to 24 hrs. Primary human microvascular endothelial cells (HMVECs), growth medium [Medium 131 with 5% microvascular cell growth supplement (MVGS)] and attachment factor were purchased from Invitrogen/Cascade Biologics; HMVECs were used between passages 3–6. Viability was measured with the XTT assay and the L-ASP IC_50_ was 37 U/ml with a continuous exposure of 6 days. Clinically targeted circulating L-ASP concentrations are in the range of 0.3–3 U/ml and constituted the treatment range used.

### Capillary-like tube formation assay

Capillary tube formation was performed *in vitro* on Matrigel [34] in the presence or absence of L-ASP over an 18–24 hrs period. High power fields were digitized at 20X magnification, and total tube length (μm) from four equal random areas per chamber was measured. A conversion factor of 0.62 μm per pixel was applied (Nikon Imagine Software BR 2.3; Nikon, Melville, NY, USA).

### Invasion assay

The HMVECs were plated with or without L-ASP in growth medium supplemented with 5% MVGS and 50 ng/ml VEGF_165_ for 16 hrs. Cells were harvested, then subjected to invasion using serum-free medium with VEGF_165_ 50 ng/ml with or without 5%MVGS as attractant. Serum-containing medium was used for ovarian cancer cell invasion. Filters were fixed, stained and invaded cells in four random microscopy fields were counted with correction for spontaneous background invasion.

### Zymography

Zymography was performed as described using conditioned medium from HT1080 cells as the control [35]. HMVECs were pre-treated with L-ASP for 20 hrs then conditioned in MVGS-free medium containing L-ASP for 24 hrs. Quantity was assessed over replicate gels using Image Quant v5.2 (Molecular Dynamics, Sunnyvale, CA, USA).

### Adhesion assays

Cell adhesion to defined matrices or antibodies examined cell-matrix interactions. Adhesion molecule (2 μg/ml) or antibody (2 μg/ml) were pre-bound on 96-well high binding plates (CoStar, Corning, Big Flats, NY, USA) at 37°C for 60 min., then blocked with 0.1% fatty acid-free BSA in PBS for 60 min. HEYA8 or HMVEC cells were pre-treated with L-ASP and incubated for 60 min. Heterotypic cell adhesion was performed as described by [14, 36]. Either tumour cells or HMVECs were pre-treated with L-ASP or VEGF_165_ in growth medium for 16 hrs prior to fixation [14, 36]. In both assays, non-adherent cells were washed off gently with PBS and adherent cells stained and quantified by XTT assay.

### Immunofluorescence and flow cytometry

Cells were grown on cover slips and treated with L-ASP, then washed and fixed, permeabilized (for IF) and stained [37]. The cytoskeleton was visualized by incubation with rhodamine-phalloidin (Invitrogen, Carlsbad, CA, USA). Cells were counterstained with DAPI (Vector laboratories, Burlingame, CA, USA) and fluorescence examined using a Zeiss LSM 510 confocal microscope (Carl Zeiss Inc, Thornwood, NY, USA) using a Nikon Plan Apo 60×/1.4 oil immersion objective. In some cases, a series of *Z*-plane optical sections were taken through the depth of the cells with a thickness of 1 μm at intervals of 0.2 μm. Expression of cell surface protein in non-permeabilized cells was assessed by flow cytometry according to manufacturer's protocol (Cell Signaling, Danvers, MA, USA). Paraformaldehyde (1%) -fixed cells were incubated with anti-sialyl Lewis^x^ (sCD15) or E-selectin (1:50; Calbiochem, San Diego, CA, USA) and visualized with Alexa 488-conjugated secondary antibodies (1:200; ThermoScientific, Rockford, IL, USA) by FACS Calibur flow analysis (BD Biosciences, San Jose, CA, USA). Data were analysed using FlowJo (TreeStar Inc, Palo Alto, CA, USA).

### Immunoblot

Cell lysates were prepared with modified RIPA lysis buffer and subjected to immunoprecipitation and immunoblotting [37]. Lysates underwent no more than one freeze/thaw cycle. All antibodies were from Cell Signaling with the exception of LC-3 (Novus Biologicals, Redmond, WA, USA) and anti-phospho-FAK (Tyr397; Millipore, Billerica, MA, USA).

### Statistical approaches

Unless otherwise indicated, unpaired Student's *t*-test was used to examine statistical relationships against vehicle control. Results are presented as mean ± S.E.M. of at least three replicate experiments, including gels, unless otherwise specified.

## Results

### L-ASP inhibits *in vitro* capillary tube formation and vascular remodelling

**Fig 1:**
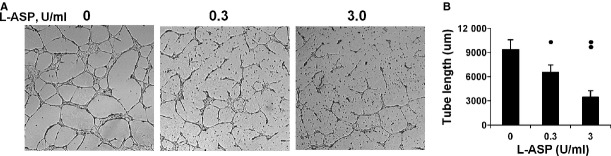
L-ASP inhibits tube formation on Matrigel. L-ASP exposure inhibited HMVEC differentiation into tubes *in vitro* in a dose dependent fashion. (A) Representative fields. (B) Cumulative tube length in 4HPF/well for replicate experiments (*P < 0.001, ** *P* < 0.005).

Vascular tube formation is approximated experimentally using an *in vitro* Matrigel substratum assay [34]. The concentrations and time of exposures used for these and all other experiments were outside the L-ASP toxicity range as described in Methods. L-ASP pre-exposed HMVECs were plated onto a Matrigel-coated chamber in the presence of L-ASP. Untreated HMVECs formed well organized tube-like structures (Fig. 1A). L-ASP 0.3 U/ml, a clinically attainable concentration, resulted in a marked reduction in the number and complexity of tube structures. Greater attenuation was observed at higher concentrations. The average summated length of capillary tubes was significantly reduced with L-ASP incubation (*P* ≤ 0.005; Fig. 1B), indicating that L-ASP inhibits morphogenesis of endothelial cells into capillary-like tubes.

### L-ASP inhibits invasion of both, but differentially inhibits attachment, of HMVEC and ovarian cancer cells

#### Inhibition of invasive behaviour of microvascular endothelial cells

**Fig 2:**
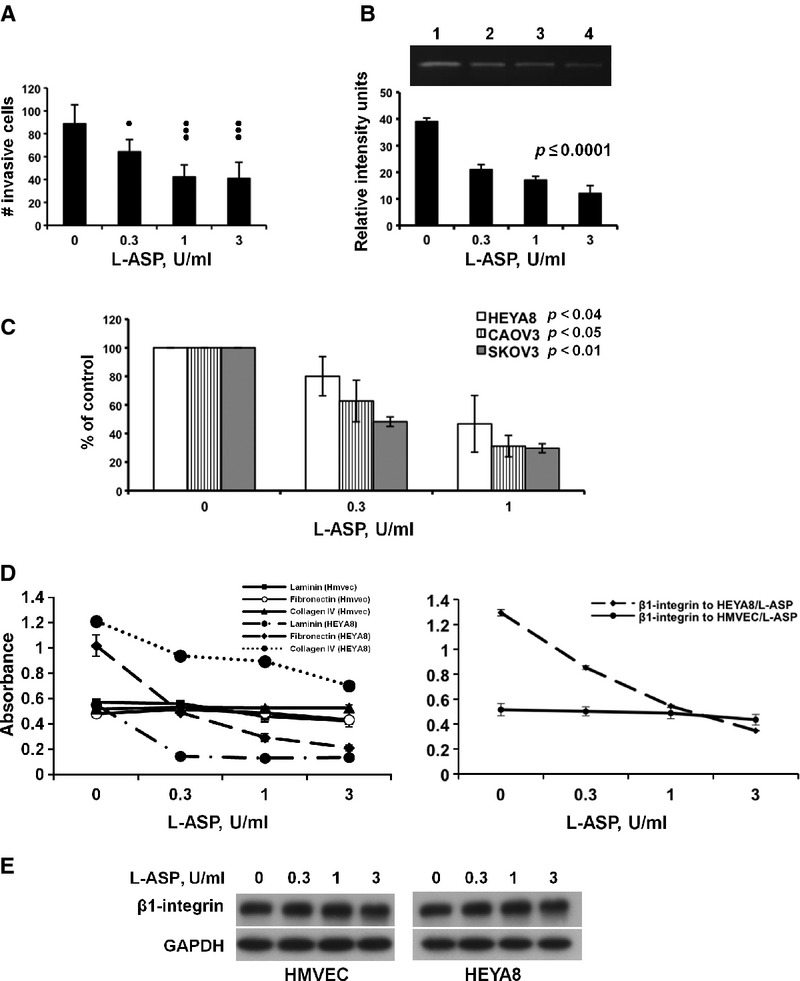
L-ASP inhibits invasion and causes differential matrix-attachment of HMVEC and ovarian cancer cells at physiologically attainable concentrations. (A) Dose-dependent inhibition of HMVEC invasion. HMVEC cells treated with and continuously exposed to L-ASP were subjected to invasion towards VEGF_165_ in complete medium; *P* ≤ 0.004. (B) L-ASP inhibits HMVEC MMP2 quantity and activity. HMVEC serum-free CM was collected and examined by zymography (quantified replicate zymograms, S.E.M., *P* ≤ 0.0001; inset, representative gel). (C) L-ASP inhibits invasion of ovarian cancer cells. Ovarian cancer cells treated with and continuously exposed to L-ASP were subjected to invasion towards serum. Replicate experiments and S.E.M. are presented. HEYA8, *P* ≤ 0.04; CAOV3, *P* ≤ 0.05; SKOV3, *P* ≤ 0.01. (D) Dose-dependent inhibition of HEYA8 but not HMVEC attachment to ECM glycoproteins. Pretreated cells were allowed to attach to indicated substratum proteins (left panel: HEYA8, dashed lines, *P* ≤ 0.0002; HMVEC cells, solid lines) or to a lawn of anti-ß1 integrin antibody (right panel, HEYA8 cells *P* = 0.02). (E) L-ASP exposure does not change expression of ß1-integrin in either cell type, shown by immunoblot.

Normal vessel remodelling is one of the several examples of physiological invasion [17]. Thus, we examined inhibition of HMVEC invasion by L-ASP. A statistically significant reduction in number of invaded HMVEC cells was observed in a dose-dependent fashion (*P* = 0.004; Fig. 2A). Breakdown of extracellular matrix by MMP-2 promotes physiologic invasion and capillary remodelling of ECs. The quantity and activity of MMP-2 in HMVEC conditioned medium was markedly reduced with increased dose of L-ASP (*P* < 0.0001; zymogram inset; Fig. 2B).

#### L-ASP inhibits ovarian cancer invasion

The concentrations and time of exposures used for all ovarian cancer cell experiments were outside the L-ASP toxicity range. L-ASP was maintained during invasion towards the serum-containing medium chemoattractant. Invasion was significantly decreased in all ovarian cancer cell lines (*P* ≤ 0.05, *P-*values were determined on experimental raw data; Fig. 2C). Thus, L-ASP inhibits both physiological invasion of vascular remodelling and malignant invasion of the ovarian cancer cells.

#### L-ASP inhibits ß1 integrin-mediated cell-ECM attachment and signalling

The invasiveness of ECs and tumour cells is regulated by interaction with the ECM through integrins, where the ß1subunit is most common. The ß1subunit has been shown to require glycosylation for activity [29, 30]. HMVEC and HEYA8 cells pre-treated with L-ASP were allowed to bind to substratum coated with laminin, fibronectin and collagen IV. No reduction in HMVEC adhesion to ECM proteins was observed; in contrast, adhesion of HEYA8 cells to laminin and fibronectin was strongly inhibited by L-ASP, with little effect on binding to collagen IV (*P* ≤ 0.0002; Fig. 2D, left). Consistent with this, defective adhesion was observed when L-ASP treated-HEYA8 cells were incubated on an anti-ß1 integrin antibody substratum (*P* = 0.02), with no significant change in HMVEC adhesion (Fig. 2D, right). Total integrin ß1 on the surface of all cells was unchanged over all concentrations of L-ASP tested (Fig. 2E). Thus, L-ASP may alter ß1-integrin conformation or surface oligosaccharide differentially on the ovarian cancer cells, resulting in altered adhesion.

### L-ASP alters FAK activation and stress fibre organization

**Fig 3:**
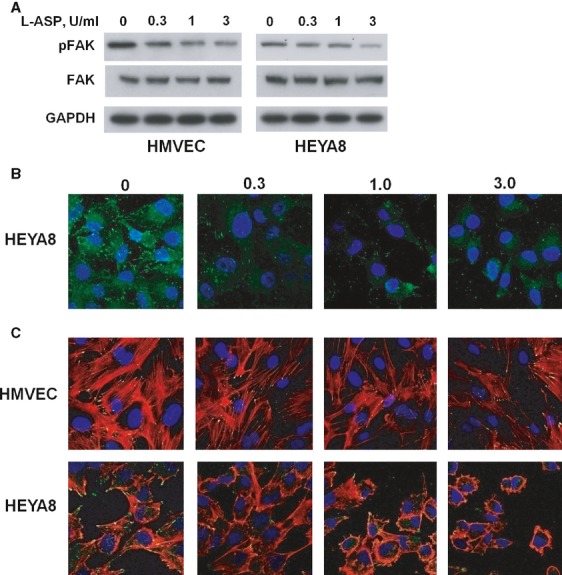
L-ASP alters FAK activation and stress fibre organization. (A) L-ASP exposure reduces p^397^Y-FAK. The dose-dependent reduction is less in HMVEC (left panel) than in HEYA8 cells (right). (B) Dose dependent reduction in HEYA8 focal adhesions labelled by p^397^Y-FAK immunofluorescence. The quantity and organization of focal adhesions is reduced in HEYA8 cells. (C) L-ASP treatment inhibits actin stress fibre formation in HMVEC and HEYA8 cells. HMVEC cells have reduction in quantity and thickness of stress fibres (arrows), whereas there is both a reduction in stress fibers (arrows) and reorganization of actin (arrowheads) in the HEYA8 cells.

Integrin-ECM interactions result in activation of FAK *via* autophosphorylation at ^397^Y [38]. L-ASP exposure reduced p^397^Y-FAK in both cell types without affecting total FAK (Fig. 3A). The lack of change in quantity of HMVEC ß1-integrin suggests that dysregulation of FAK may be due to other effects of L-ASP. Focal adhesions are the outside-in integrin-mediated signalling bodies that stimulate formation of actin stress fibres. Immunofluorescence imaging of HEYA8 cells confirmed reduced matrix-cell signalling by showing reduction in p^397^Y-FAK-labelled focal adhesions (Fig. 3B). L-ASP reduced number and thickness of the actin stress fibres in HMVEC cells. Stress fibres were reduced in quantity and associated with a change in actin organization and cell shape in the HEYA8 ovarian cancer cells (Fig. 3C, top and bottom respectively); the effect on the ovarian cancer cells shape and size and degree of stress fibres appears stronger. These results suggest that L-ASP may selectively modify extracellular ß1-integrin in ovarian cancer cells over HMVEC causing inhibition of integrin engagement, and reduced adhesion to matrix and altered actin reorganization.

### Inhibition of heterotypic adhesion of ovarian cancer cells to ECs

**Fig 4:**
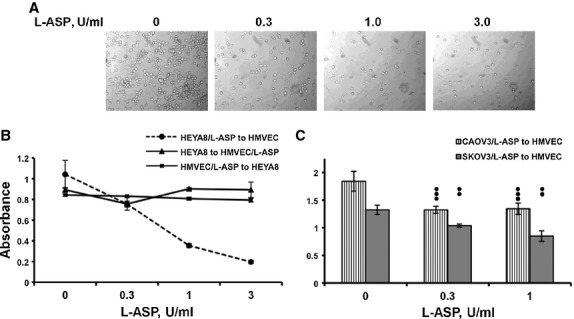
Heterotypic adhesion of tumour cells to HMVEC cells is reduced by L-ASP treatment. Treated HEYA8 cells are unable to bind to a monolayer of HMVEC cells in a dose-dependent fashion. (A) Representative adhesion fields. (B) Quantification showing only treatment of HEYA8 cells results in inhibition of heterotypic binding (dotted line; 0.3 U/ml, *P* < 0.05; 1.0 U/ml, *P* < 0.01; 3 U/ml, *P* < 0.006). (C) Similar loss of heterotypic adhesion is seen with treatment of CAOV3 (*P* ≤ 0.001) and SKOV3 (*P* ≤ 0.01) ovarian cancer cells.

Cells receive survival signals *via* ECM- and cell-cell mediated attachment. Heterotypic cell-cell adhesion is an early step in tumour dissemination and has been shown to involve glyco- and sialyl-proteins [4, 25]. We therefore asked if L-ASP effects extended to cell-cell adhesion. L-ASP treated ovarian cancer cells had reduced adhesion to an untreated, fixed (non-viable) HMVEC monolayer. Reduction in heterotypic cell adhesion occurred only when tumour cells were exposed to L-ASP (Fig. 4A,B; *P* < 0.01–0.05). No effect of L-ASP treatment of HMVEC was seen whether treated HMVEC cells were immobilized and fixed for tumour cell adhesion (Fig. 4B, triangles) or applied viably to immobilized, fixed ovarian cancer cells (HEYA8, Fig. 4B squares; SKOV3, CAOV3 cells, Fig. 4C; *P* ≤ 0.01). SKOV3 and CAOV3 cells were examined up to 1 U/ml to corroborate the effects of the HEYA8 and to avoid broaching toxic concentrations. These data indicate that L-ASP alters cell-cell adhesion in a unidirectional fashion, implicating alteration of a key cell surface component of the ovarian cancer cells required for attachment to HMVECs.

### Cell surface sLe^x^ is reduced by L-ASP treatment

**Fig 5:**
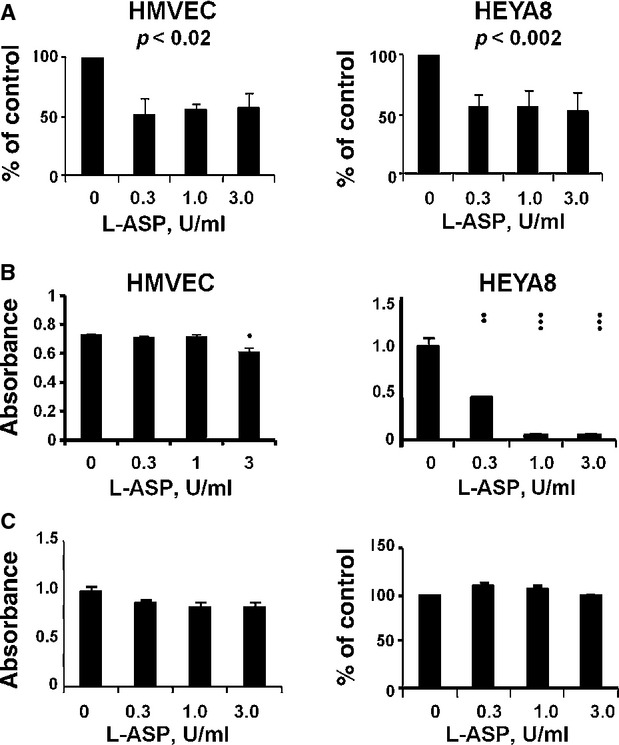
L-ASP alters surface sLe^x^ expression and sLe^x^-requiring responses. (A) Reduction in sLe^x^ expression, mean ± S.E.M. S.E.M. (HMVEC, *P* < 0.02; HEYA8, *P* < 0.002). (B) L-ASP exposed HMVEC were not and HEYA8 cells were inhibited in a dose-dependent fashion in binding to a lawn of anti- sLe^x^. (1 dot, *P* = 0.02; 2 dots, *P* ≤ 0.01; 3 dots, *P* ≤ 0.001). (C) L-ASP does not affect E-selectin. Pretreated HMVEC cells have no reduction in their binding to immobilized anti-E-selectin (left panel) nor is their expressed E-selectin affected (right panel).

Sialylation of Le^x^ is a common regulatory event required for morphogenesis of ECs into capillary tubes, activation of ß1 integrin and for heterotypic cell-endothelial cell binding [16, 29, 39]. L-ASP treatment significantly reduced surface sLe^x^ expression in HMVEC and HEYA8 cells measured by flow cytometry (Fig. 5A; *P* ≤ 0.02 and <0.002 respectively). Reduced surface sLe^x^ was associated with decreased binding of treated HEYA8 cells to the anti-sLe^x^ substratum (Fig. 5B; *P* ≤ 0.01), but not of treated HMVEC cells. The dichotomous behaviour with a dose dependent change in binding to sLe^x^, but a stable reduction in quantity of sLe^x^ is a consistent observation. sLe^x^ on the surface of solid tumour cells serves as a major ligand for E-selectin on ECs [14, 36]. L-ASP did not alter the ability of HMVEC to bind to an anti-E-selectin substratum (Fig. 5C, left panel); likewise, we found no alteration in HMVEC cell surface E-selectin quantity (Fig. 5C, right panel). The L-ASP mediated reduction in HMVEC surface sLe^x^ did not alter their E-selectin adhesive function. Thus, L-ASP inhibition of sLe^x^ expression on ovarian cancer cells unilaterally inhibits binding to sialylated integrin or HMVEC cell surface E-selectin.

### Induction of autophagy by L-ASP

**Fig 6:**
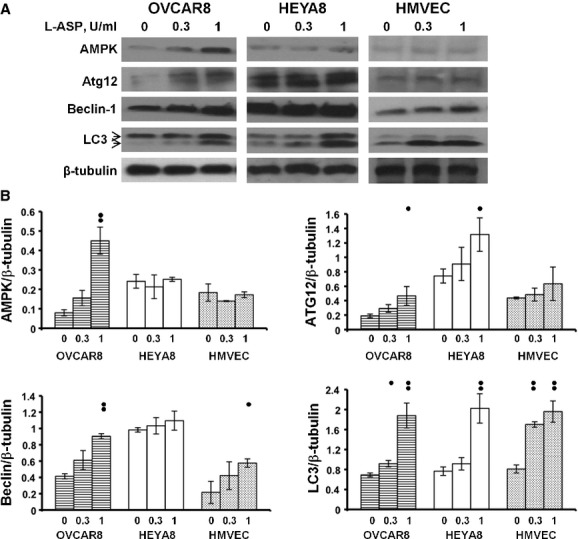
L-ASP induces autophagy in HMVEC and ovarian cancer cells. Exposure to L-ASP in the presence of serum results in induction of AMPK, ATG12, beclin-1, and LC3 cleavage in OVCAR8, HEYA8, and HMVEC cells. (A) Representative immunoblots. LC3 cleavage from LC3-I to LC3–II is shown with arrows. (B) Quantification of replicate immunoblots, mean ± S.E.M., one dot, *P* < 0.01; two dots, *P* ≤ 0.001.

The L-ASP inhibits glycosylation-associated events necessary for ovarian cancer survival and dissemination at concentrations and durations that did not cause cell death. However, it has been shown previously to inhibit ovarian cancer proliferation [12, 13]. Autophagy is a mechanism through which the cell attempts to protect itself from injury and death by shutting down its environmental interactions and using its internal resources for survival but may remain functional [40–42]. Exposure to L-ASP for 16 hrs caused no reduction in mitochondrial respiration by XTT assay (Fig. 1) but did activate autophagy pathways both in the presence (Fig. 6) or absence of serum (not shown). L-ASP concentrations only up to 1 U/ml were used to minimize transition from autophagy to apoptosis. Induction of AMPK, ATG12, beclin-1 and/or cleavage of LC3 occurred at physiologically attainable L-ASP concentrations (0.3–1 U/ml) in two ovarian cancer cell lines and the primary culture HMVEC cells; similar beclin induction results were observed in CAOV3 cells as well. These results indicate that both microvascular cells and ovarian cancer cells are susceptible to potential L-ASP-associated injury during conditions where there is reduction in invasive and angiogenic activity.

## Discussion

Focal genetic driving events have not been identified for the bulk of high-grade serous ovarian cancers TCGA [43]; thus, successful intervention requires a broader look at the cellular and molecular events involved in supporting and sustaining its growth and dissemination. We and others have demonstrated a close link between angiogenesis and ovarian cancer at the correlative, prognostic, predictive and clinical levels and numerous clinical trials have examined the role of angiogenesis as a molecular target in ovarian cancer [31, 32]. Active vascularization has been confirmed to be an important molecular target in the tumour microenvironment of ovarian cancer, requiring cell-cell and cell-matrix interactions for success [17]. We have sought new clinically translatable mechanisms with which to modulate angiogenesis in ovarian cancer.

Many agents studied and applied early in the anti-neoplastic era were examined selectively in a leukaemia background. Some, such as L-ASP, went on to be successful anti-leukemic agents and had no or little additional examination in solid tumours after limited initial success. The role of asparaginase in ALL has been attributed to its anti-metabolic activity, essentially removing adequate amounts of the necessary amino acid, asparagine, required for the rapid growth, metabolism and protein glycosylation in ALL cells [8, 10]. It was proposed early on that an important mechanism of action of L-ASP is the inhibition of *de novo* production of asparaginyl-*N*-glycoproteins at the post-Golgi level [8, 9]. CD15, a cell-surface glycan consisting of the Gal-ß(1–4)-(Fuc-a(1–3))-GlcNAc trisaccharide, is differentially recognized when sialylated [44, 45]. Sialyl-CD15 (sCD15) recognizes the sLe^x^ epitopes that bind E-selectin and facilitate heterotypic cell adhesion. CD15 sialylation and sialidase activity regulating CD15 expression on human myeloid cells was shown to regulate their differentiation [44]. It is thus possible that L-ASP may promote myeloid differentiation during leukaemia therapy by inhibition of sLe^x^. Likewise, the reduction in HMVEC differentiation activity in our *in vitro* models in response to L-ASP exposure may be a function of interruption of sLe^x^/E-selectin differentiation signalling.

Asparagine synthetase, the enzyme responsible for synthesis of asparaginase, may be differentially expressed in tumour cells contributing to poor L-ASP activity. Low expression of ASNS was identified in ovarian cancer cell lines in which an inverse response between ASNS quantity and susceptibility to L-ASP was demonstrated [12, 13]. We suggest that the functional activities of L-ASP would relate to its anti-metabolic/glycosyl regulatory activities. Reduction of glycosylation-associated activation events should and did reduce angiogenesis and invasive behaviour. This would lead to a locally non-permissive microenvironmental cancer milieu while not altering the normal physiology. Our findings confirming this provide an argument for clinical examination of L-ASP in ovarian cancer.

Both endothelial and ovarian cancer cells had an approximately 50% reduction in surface sLe^x^ expression with L-ASP exposure. Only the ovarian cancer cells were inhibited by L-ASP in binding to anti-sLe^x^, indicating a unidirectional process. The lack of reduction in HMVEC E-selectin, the sLe^x^ ligand, further supports the one-sidedness of the finding and suggests that L-ASP modulates the permissiveness of the tumour microenvironment, a hypothesis that may apply also to ALL in the bone marrow [11, 46]. The dichotomy between reduction in surface sLe^x^ and the binding of the HEYA8 cells to it may be due to the quality and extent of sialylation. A key enzyme in sialylation is β-galactoside α2,6-sialyltrans-ferase (ST6Gal-I). This enzyme has been shown to be upregulated in ovarian cancer cells promoting invasiveness through sialylation of β_1_ integrin [39]. Similar increase in attachment to basement membrane collagen IV was associated with ST6Gal-I enzyme activity in breast cancer cells [47]. Tumours induced in MMTV-*ST6Gal-I* null mice have more normal breast differentiation, decreased activation of FAK and when genetically complemented, have restored ß_1_-integrin signalling [48]. Our findings of reduction in functional ß_1_-integrin and inhibited binding to β_1_-integrin in response to L-ASP are consistent with these mechanisms.

The lack of reduction in cell-cell attachment when the ECs were exposed to L-ASP can be explained by the sidedness of glycosylation-requiring adhesion events. ECs are activated in response to extracellular matrix protein binding, growth factors and *via* E-selectin in heterotypic interactions with tumour cells [14, 16]. Each can result in induction of motility, invasion and proliferation. E-selectin binding is specific to Lewis^A/X^-containing ligands [16]. VEGF_165_ is a glycosylated isoform of the VEGF protein and is a critical stimulant of endothelium that is produced by the tumour microenvironment, and most or all ovarian cancer cells. Affinity and response to the different VEGF isoforms is different, with VEGF_165_ being one of the most potent. Altered recognition of this glycosylated product may be another mechanism through which L-ASP alters microenvironment activity. VEGF_165_ alone is insufficient to induce and sustain HMVEC or HUVEC tube formation on Matrigel in our hands (Kohn, unpublished data). L-ASP incubation did not reduce VEGF production by the ovarian cancer cell lines, as measured by specific ELISA using conditioned medium (not shown).

We showed that L-ASP reduced physiological and malignant invasive events at concentrations and durations of exposure that were non-toxic. This growth independence from sLe^x^ has also been described in colon cancer cells where specifically selected high and low sLe^x^ expressors were found to have the same proliferative capacity but low sLe^x^ expression was associated with reduced invasion [7]. We then made two seminal observations: (1) adhesive behaviour was unidirectional with the primary effects of L-ASP against the ovarian cancer cells blocking adhesion to HMVEC cells and glycosylated extracellular basement membrane proteins; and (2) L-ASP induced autophagy in both HMVEC and ovarian cancer cells, where the earliest signs were observed as early as 24 hrs. Loss of attachment is known to induce apoptosis through anoikis [19] but this may be forestalled if the cells enter autophagy first [49, 50]. Autophagy is a mechanism through which cells rely on their internal resources to ride out a storm, such as exposure to an anti-metabolite like L-ASP [40, 42, 51]. As expected, some variance in the degree of autophagy and concentration sensitivity was observed. Induction of autophagy concomitantly with loss of invasive behaviour has been seen with many targeted and anti-angiogenic agents [52, 53] and could explain the lack of cytotoxicity we observed.

These findings elucidate a novel paradigm for L-ASP mechanism of action. The reduction in sialylation needed for integrin activation and engagement, and heterotypic cell-cell adhesion alters the balance of interaction and cell signalling in the local tumour microenvironment. Heterotypic adhesion of ovarian cancer cells also occurs to the peritoneal mesothelium, facilitating shedding and subsequent dissemination. This may occur in part through the mesothelin/CA125 interaction, another glycosylated process [54]. This may be an adequate single agent therapeutic in rapidly growing and angiogenesis-requiring ALL. There may be therapeutic activity of L-ASP in the angiogenic setting in solid tumours, although it may require combination therapy to realize its full potential. The pegylated asparaginase, pegaspargase, now in use provides an alternative intravenous formulation with which to further examine L-ASP behaviour. A pilot phase II trial of pegaspargase in ovarian cancer is open in our institution (NCT01313078) and includes translational angiogenesis and glycosylation endpoints to illustrate this potential novel mechanism of action.
